# Genetically proxied inhibition of kidney function pathways and increased risk of type 2 diabetes in Africans: A Mendelian randomization study

**DOI:** 10.1177/00368504251338631

**Published:** 2025-06-17

**Authors:** Abdoulaye Diawara, Mariam Traore, Oudou Diabaté, Christopher Kintu, Ali Awadallah Saeed, Julianah Ore Abiola, Cheickna Cisse, Kassim Traore, Mamadou Wele, Oyekanmi Nash, Seydou O. Doumbia, Talib Yusuf Abbas, Jeffrey G. Shaffer, Mahamadou Diakité, Segun Fatumo, Opeyemi Soremekun

**Affiliations:** 1 African Center for Excellence in Bioinformatics of Bamako (ACE-B), University of Sciences, Techniques and Technologies of Bamako (USTTB), Bamako, Mali; 2 African Computational Genomics Research Group, MRC/UVRI and LSHTM Uganda Research Unit, Entebbe, Uganda; 3 Laboratory of Research in Microbiology and Microbial Biotechnology (Laborem-Biotech), University of Sciences, Techniques and Technologies of Bamako (USTTB), Bamako, Mali; 4 University of Sciences, Techniques and Technologies of Bamako, Bamako, Mali; 5 Faculty of Pharmacy, Department of Pharmacology, National University-Sudan, Khartoum, Sudan; 6 Centre for Genomics Research and Innovation (CGRI), National Biotechnology Development Agency (NABDA), Abuja, Nigeria; 7 Department of Biochemistry and Molecular Biology, University of Sciences, Techniques and Technologies of Bamako (USTTB), Bamako, Mali; 8 Department of Biochemistry, College of Osteopathic Medicine, Duquesne University, Pittsburgh, Pennsylvania, USA; 9 Department of Public Health, Faculty of Medicine and Odontostomatology (FMOS), University of Sciences, Techniques and Technologies of Bamako (USTTB), Bamako, Mali; 10 Burhani College, Mumbai, India; 11 25812 Tulane University School of Public Health and Tropical Medicine, New Orleans, Louisiana, USA; 12 Precision Healthcare University Research Institute, Queen Mary University of London, UK; 13 London School of Hygiene and Tropical Medicine, London, UK; 14 Medical Research Council, Uganda Virus Research Institute, Entebbe, Uganda; 15 Molecular Bio-computation and Drug Design Laboratory, School of Health Sciences, University of KwaZulu-Natal, Durban, South Africa

**Keywords:** diabetes, kidney disease, estimated glomerular filtration rate, Mendelian randomization, Africa

## Abstract

**Objective:**

To investigate the causal relationship between genetically predicted inhibition of specific kidney function drug targets and the risk of type 2 diabetes (T2D) in African populations using Mendelian randomization (MR).

**Methods:**

We used MR, a genetic proxy approach, and utilized genome-wide association study data from African participants. This assessed the causal relationship between genetically predicted inhibition of specific pathways and T2D risk. The analysis was conducted using TwoSampleMR package implemented in R.

**Results:**

We found that inhibiting the vascular endothelial growth factor A (VEGFA) and Ras homolog enriched in brain (RHEB) was significantly linked to T2D risk in Africans (OR 2.66, 95% CI 1.34–3.78, p = 0.0017 and OR 2.25, 95% CI 1.34–3.28, p = 0.0010, respectively). Conversely, there was no evidence that solute-like carrier family 22 member A2 or claudin-14 were associated with an increased risk of T2D (OR = 0.95, 95% CI 0.61–1.48; OR = 1.56, 95% CI 0.71–2.20, respectively).

**Conclusions:**

Insight from this study could potentially mean that some of the drugs that are used for treatment of kidney diseases involving VEGFA and RHEB may potentially increase the risk of developing T2D among Africans. This highlights how it is critical to consider drug–drug interaction in kidney diseases in Africa.

## Introduction

Type 2 Diabetes (T2D) also known as non-insulin-dependent diabetes mellitus or maturity onset diabetes is a long-term disease that results from elevated blood glucose levels due of reduced sensitivity of the cells to insulin action or inadequate insulin release by the pancreatic beta cells.^1,2^ It is a major global health concern and currently affects low- and middle-income countries (LMICs), including those in Africa in a relatively higher proportion.^3–6^ Importantly, diabetes is a key risk factor for chronic kidney disease (CKD) and a major complication of T2D, amplifying the risk of cardiovascular events and mortality.^7,8^ Permanent hyperglycemia leads to widespread inflammation in the body, including the kidneys. Immune cells are attracted to the kidneys and become very active, releasing cytokines in this inflammatory environment, further aggravating the inflammation.^9,10^ This chronic inflammation leads to kidney cell damage and progressive loss of kidney function. Although there are several treatment options, including diabetes drugs, access to these treatments may be limited in low-resource regions.^11–13^

This study aims to assess the relationship between the inhibition of specific drug targets that treat renal failure and the development of T2D in African populations, using Mendelian randomization (MR).

MR is an epidemiological method that uses natural genetic variations as instrumental variables (IVs) to estimate the causal effect of an exposure on an outcome.^14^ By using genetic variants, MR offers several advantages over traditional observational studies.^15,16^ It can help overcome some of the limiting factors of observational studies, such as confounding and reverse causality, and provide stronger evidence of causality.^16^

Using MR, we can investigate the causal link between inhibition of these drug targets and the risk of T2D, providing useful information about the potential risks associated with these treatments. A preliminary version of this work has been previously published as a preprint on Research Square (DOI: https://doi.org/10.21203/rs.3.rs-3956597/v1).^17^

## Methods

### Study design

**Figure 1.**
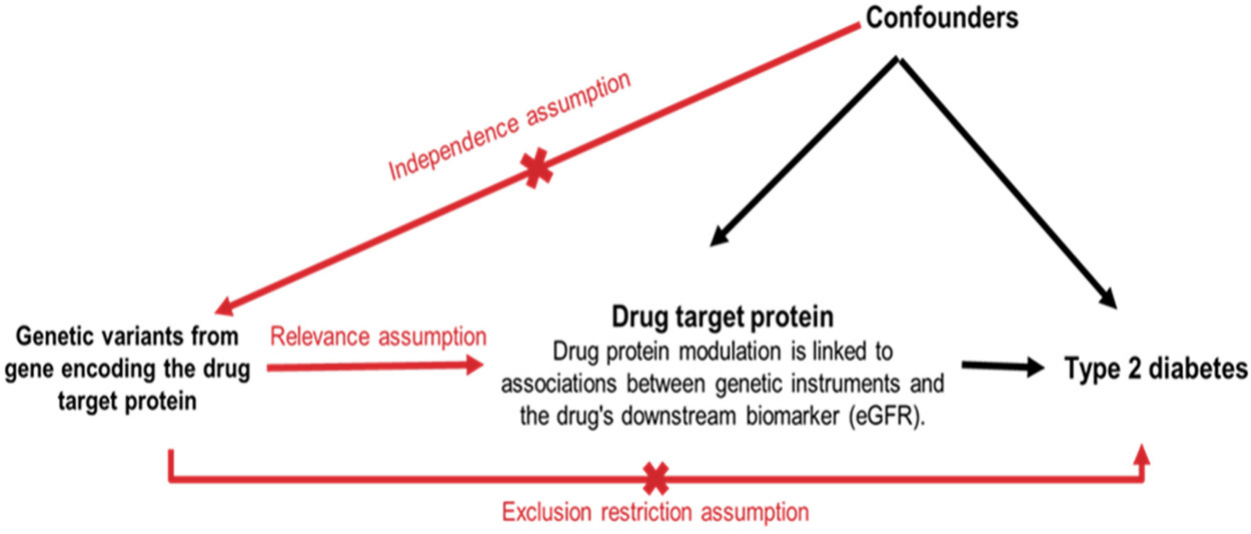
Assumptions of Mendelian randomization for assessing the causal effect of kidney disease drug targets on type 2 diabetes.

This study employed a two-sample MR design to investigate the potential association between inhibiting drug targets for kidney disease and the development or progression of T2D in African populations^18^ (Figure 1). The analysis was conducted using R version 4.3.0 and the TwoSampleMR package.^18^

Figure 1 explains the key assumptions underlying the use of MR to assess the causal effect of kidney disease drug targets on T2D. Genetic variants within genes encoding proteins targeted by kidney disease drugs serve as IVs in the MR analysis. The drug target protein corresponds to the protein directly influenced by the drug. T2D is the outcome of interest in this study. The Relevance Assumption posits that the selected genetic variants are truly associated with the function or expression of the targeted protein. The Independence Assumption asserts that the selected genetic variants are not associated with any other factors that could directly or indirectly influence T2D, besides their effect on the drug target protein. The Exclusion Restriction Assumption posits that the genetic variants only influence T2D through their effect on the drug target protein and do not exert any other independent effects on T2D. Confounders represent unmeasured or measured factors that could influence both the drug target protein and T2D, potentially leading to biased results if not adequately accounted for in the analysis.

### Selection of drug targets and genetic instruments

#### Drug target identification

We identified potential drug targets relevant to kidney function by mining resources such as DrugBank and relevant databases, along with leveraging data from published studies.

### Gene selection

**Figure 2.**
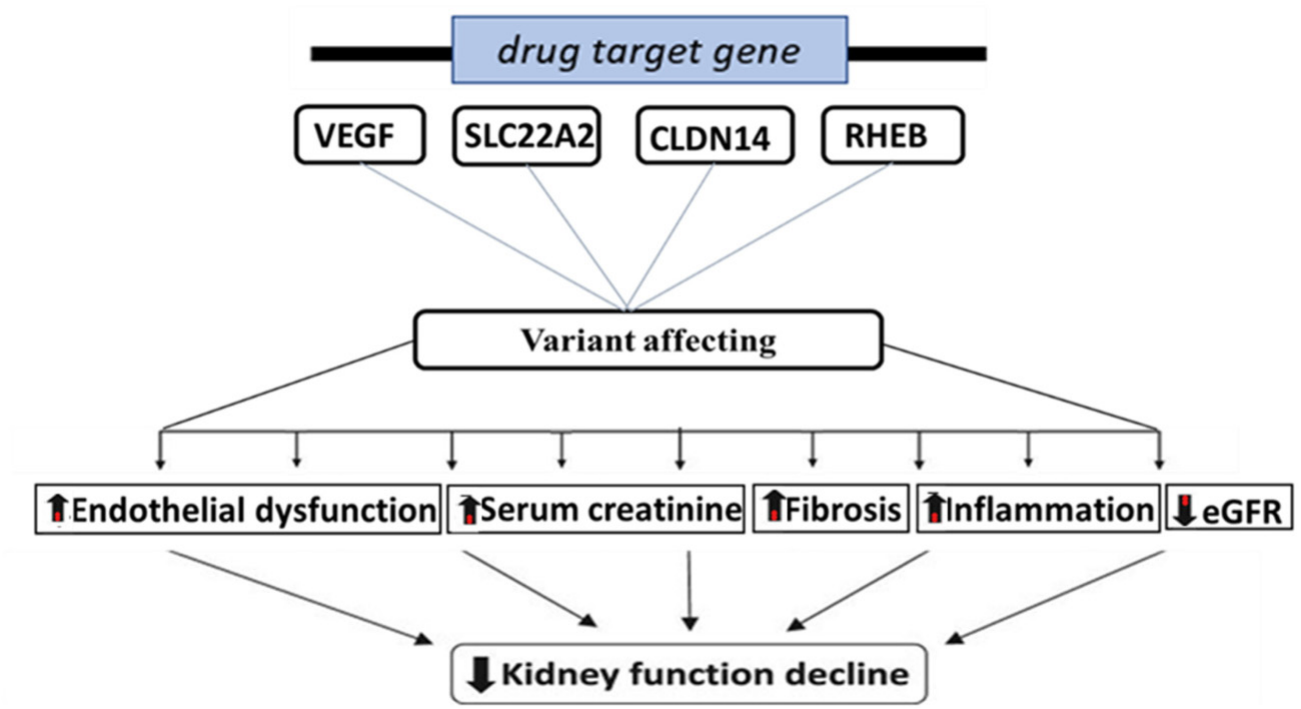
Pathways linking genetic variation in drug targets to kidney function decline.

Based on their established roles in pathways related to endothelial dysfunction, serum creatinine, fibrosis, inflammation, the estimated glomerular filtration rate (eGFR), and decline in renal function, we selected the following genes as potential drug targets in the context of T2D and associated renal complications: vascular endothelial growth factor (VEGF), solute-like carrier family 22 member A2 (SLC22A2), claudin-14 (CLDN14), and Ras homolog enriched in brain (RHEB) (Figure 2). RHEB was chosen precisely due to its responsibility as a control activator of mTORC1 signaling, a source regulator of cell growth, proliferation, and glucose metabolism.^19,20^

*Figure 2 illustrates how variations in these VEGF, SLC22A2, CLDN14, and RHEB genes can affect various physiological processes, such as endothelial dysfunction, increased serum creatinine, fibrosis, and inflammation, ultimately leading to a decline in kidney function.*

### Genetic instrument selection

We then selected single-nucleotide polymorphisms (SNPs) located within or near (±300 kb) of these genes that were previously associated with eGFR at genome-wide significance (p < 5 × 10^−8^). These SNPs were used as IVs to estimate the causal effect of inhibiting the drug target on T2D risk.

### Data sources

#### Exposure (eGFR)

Genetic associations of eGFR were obtained from a meta-analysis of large genome-wide association studies (GWAS) conducted in individuals of African ancestry. The data sources included the Million Veteran Program (MVP),^21^ the Chronic Kidney Disease Genetics Consortium of African descent,^22^ and the UK Biobank.^23^

#### Outcome (T2D)

Summary statistics for T2D in Africans were obtained from a meta-analysis of two GWAS studies: the African American Diabetes Mellitus (AADM) study and Durban Diabetes Study (DDS).^24^ The AADM study included 2707 participants of African descent, while the DDS focused on a population-based sample of Zulu adults with T2D residing in Durban, South Africa.^24^

### Statistical methods

#### Single-nucleotide polymorphism clumping and f-statistics

To ensure the selected SNPs were independent, we performed clumping with a linkage disequilibrium threshold of r < 0.1, using the 1000G African reference panel.^25^ The F-statistics were calculated for each SNP to assess instrument strength, and only SNPs with an F-statistic > 10 were retained for further analysis.^26^

#### Allele harmonization

Allele harmonization was performed to guarantee that the effect allele for each SNP was consistent across the eGFR and T2D GWAS datasets, avoiding inconsistencies in effect direction. Palindromic SNPs (C/G or A/T) with an allele frequency between 0.42 and 0.58 were excluded due to potential bias introduced by ambiguous strand information.^27^

#### Mendelian randomization analysis

We employed a two-sample MR approach with inverse variance weighting (IVW) as the primary method for estimating the causal effect of each genetic instrument on T2D risk.^28^ For genes with fewer than two instruments, the Wald ratio method was used.

### Sensitivity analyses

To address potential biases and ensure robustness of the findings, we conducted several sensitivity analyses: MR-Egger regression: this method assesses directional pleiotropy, where a SNP influences a trait correlated with the outcome in the same direction as the exposure.^28,29^ Weighted median and weighted mode: these methods provide alternative causal effect estimates less susceptible to outliers and potential pleiotropy compared to IVW.^30^ This study was conducted in accordance with the principles of the Declaration of Helsinki (1975, as revised in 2013).

## Results

### Genetic instruments for kidney disease drug targets

We identified a set of genes encoding crucial proteins involved in various kidney disease pathways. Table 1 provides details on these genes and their functions. Table 1 also shows the number of SNPs associated with each exposure (nSNPs) and the median F-statistics with their range. RHEB exhibited the highest median F-statistic (136.84), suggesting strong genetic instruments. CLDN14 modulators had the lowest median F-statistic (34.02) among the investigated exposures (Table 1). However, the F-statistics for all genes fell within an acceptable range, indicating a minimal risk of weak instrument bias. Vascular endothelial growth factor A (VEGFA) inhibitors and SLC22A2 inhibitors both demonstrated F-statistics within a reasonable range (91.98 and 141.10, respectively), suggesting sufficient instrument strength for further analysis. These findings suggest that all identified genetic instruments are likely valid for MR analysis to assess the causal effects of targeting these specific kidney disease pathways on T2D. A detailed overview of the SNPs used as instruments is provided in Supplementary Table S1 (Table S1).

**Table 1.** Drug targets and encoding genes.

Gene	Drug class	Substance	nSNPs	DrugBank ID	Function in kidney	Median F (range)
VEGF	Anti-angiogenic agents	Bevacizumab	6	DB00112	Promotes blood vessel formation	91.98 (35.1–96.11)
SLC22A2	Organic cation transporters (OCT) inhibitors	Cimetidine	8	DB00501	Involved in reabsorption of organic cations	141.10 (41.22–127.59)
CLDN14	Tight junction modulators	Lumasiran	4	DB15935	Regulates the passage of molecules between cells	34.02 (30.08–37.03)
RHEB	mTORC1 inhibitors	Tipiracil	9	DB09343	Involved in cell growth and proliferation	136.84 (38.08–243.29)

VEGF: vascular endothelial growth factor; RHEB: Ras homolog enriched in brain; SLC22A2: solute-like carrier family 22 member A2; CLDN14: claudin-14.

### Mendelian randomization analysis

Univariable MR Analysis: We found strong evidence that genetically predicted inhibition of VEGFA and RHEB were associated with an increased risk of T2D VEGFA (OR = 2.66, 95% CI: 1.34–3.78, p = 0.001) and RHEB (OR = 2.25, 95% CI: 1.34–3.28, p = 0.001) were associated with an increased risk of T2D. In contrast, there was no statistically significant association between genetically predicted inhibition of SLC22A2 (OR = 0.95, 95% CI: 0.61–1.48, p = 0.84) or CLDN14 (OR = 1.56, 95% CI: 0.71–2.20, p = 0.84) and the risk of T2D (Table 2). The link between RHEB inhibition and increased T2D risk supports the proven role of mTORC1 signaling in glucose absorption and insulin sensitivity.^31^

**Table 2.** Univariable IVW Mendelian randomization results.

Exposure	Outcome	BETA	SE	95% CI	p-value
RHEB inhibitors	T2D	0.815	0.815	(1.347, 3.289)	0.001
CLDN14 modulators	T2D	0.940	0.940	(0.712, 2.207)	0.149
VEGFA inhibitors	T2D	0.981	0.981	(1.843, 3.335)	0.001
SLC22A2 inhibitors	T2D	−0.044	−3.007	(0.615, 1.483)	0.841

T2D: type 2 diabetes; VEGFA: vascular endothelial growth factor A; RHEB: Ras homolog enriched in brain; SLC22A2: solute-like carrier family 22 member A2; CLDN14: claudin-14; IVW: inverse variance weighted.

### Sensitivity analyses

**Figure 3.**
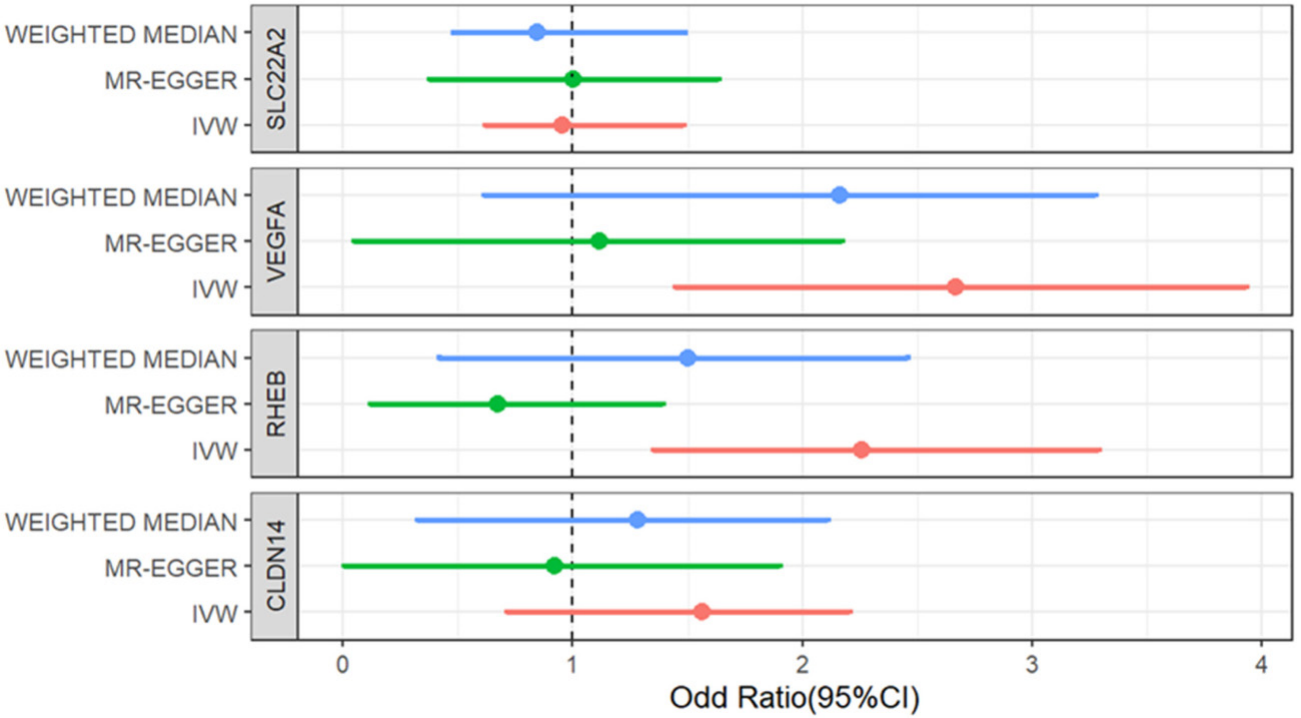
Forest plot of odds ratios for T2D risk by genetically predicted inhibition of kidney function pathways. T2D: type 2 diabetes.

To assess the robustness of the results, we conducted sensitivity analyses using the MR-Egger regression, weighted media, and weighted mode methods. . The MR-Egger intercept was not statistically significant (p = 0.194), indicating no evidence of horizontal pleiotropy. This suggests that the IVW estimates are likely unbiased. The Weighted Median and weighted mode methods yielded results reliable with the IVW estimates, further comforting the robustness of our results (Figure 3).

In addition, leave-one-out sensitivity analyses demonstrated that the results were not driven by any single genetic variant (Figure S1). To assess potential pleiotropy, we constructed funnel plots VEGFA (A) and RHEB (B), as shown in Figure S2. The proportional sharing of effect estimates around the IVW evaluation suggests the non-existence of significant pleiotropic bias. Finally, the scatter plot in Figure S3 shows the correlation between the genetic instruments for WEGFA and RHEB inhibition and their association with T2D risk, supporting further visual confirmation of the observed association.

Figure 3 presents a forest plot visualizing the odds ratios and their 95% confidence intervals for the associations between genetically predicted kidney function drug targets and T2D, estimated using the IVW, weighted median, and MR-Egger methods.

## Discussion

This research provides insights into the potential association between the inhibition of specific drug targets for kidney disease and the risk of T2D in Africans. This focus on African ancestry is crucial because genetic and environmental factors can influence both kidney disease and T2D risk.^32^ Using a two-sample MR approach, we found a significant association between inhibiting two genetic pathways (VEGFA and RHEB) and an increased risk of T2D (Table 2). Conversely, inhibiting the SLC22A2 and CLDN14 pathways showed no connection to a higher T2D risk (Figure 2).

Several medications are currently in use aimed at inhibiting VEGFA and RHEB pathways, but they are primarily intended for persons other than those suffering from T2D. Anti-VEGF therapies, particularly bevacizumab today used in cancer treatment in both solid (e.g. renal cell carcinoma) and blood-derived tumors as well ocular diseases (e.g. macular degeneration).^33,34^ These drugs work by stopping the growth of tumors or the creation of new blood vessels that nourish and protect them.^35^ In addition, mTOR inhibitors such as EVROLELIMUS and SIROLIMUS that indirectly act on RHEB signaling by inhibiting the mTOR-pathway are also used in cancer therapy (e.g. renal cell carcinoma) and organ transplantation to prevent rejections.^36,37^

Although not formally approved for T2D treatment, these drugs’ mechanisms of action suggest they are likely to have metabolic effects particularly among individuals with genetic risk factors for T2D.^31,38^ Our results provide evidence that the use of these medications in patients at risk for T2D, especially among those of African ancestry, should be monitored. Further studies are needed to investigate if genetic biomarkers, such as the SNPs delineated in this study, could be used to inform personalized treatment modifications to mitigate T2D risk among patients undergoing these therapies. The notion behind this study resonates with the potential of pharmacogenomics, the possibilities of increasing efficacy and safety in drug therapy according to a person's genetic profile.

Further research is needed to explore whether these therapies could affect T2D risk or evolution in African populations, given the findings of this study.^39^

Our results suggest that pathways specific to kidney disease may play a role in the development of T2D. The detected relationship linking genetically predicted inhibition of VEGFA and increased T2D risk align with established literature indicating that VEGF plays a fundamental role in endothelial function beyond angiogenesis.^40^ VEGF is critical for preserving vascular integrity and promoting sufficient blood flow to tissues, including adipose tissue. Impaired VEGF signaling can lead to endothelial dysfunction, decreased tissue perfusion, and ultimately contribute to insulin resistance.^40,41^ Also, the association between RHEB inhibition and increased T2D risk supports the critical role of mTORC1 signaling in controlling glucose metabolism. Various studies have supported that mTORC1 inhibition can increase insulin sensitivity and glucose metabolism in animal models of T2D.^42,43^ These results provide strong confirmation of the involvement of these pathways in the pathogenesis of T2D.

The association of genetically predicted RHEB inhibition with polygenic risk of T2D highlights the importance of mTORC1 factor (i.e. driven RHEB inhibition) in glucose homeostasis. The small GTPase, RHEB, acts as a direct activator of the mTORC1 pathway, a master regulator of cellular growth, proliferation and metabolic functions.^44^ It is important to note that mTORC1 signaling has dual effects on glucose homeostasis. Acute blockade of this pathway may increase insulin sensitivity; however, chronic downregulation can lead to metabolic awry, leading to abnormal glucose homeostasis and a greater risk of T2D.^31^

Our study acknowledge certain limitations of the relationship between renal function and the risk of T2DM in Africans but also underscores the application of MR for genetically proxied inhibition of kidney function pathways and increased and T2D risk in Africans.^3,45^ This paves the way for the development of effective, accessible, and well-tolerated therapies for T2DM and CKD in Africa, a region with significant unmet medical needs.^46^

Although the eGFR GWAS data probably did not include subjects with advanced kidney disease, including severe DKD, the T2D GWAS data (AADM and DDS) may have incorporated subjects with DKD. This is relevant because DKD could affect the genetic instruments, and the outcome potentially introducing bias or confounding. However, our analyses were primarily concerned with the causal relationship between genetically proxied inhibition of kidney function pathways and risk of T2D, rather than the progression of DKD. Future studies should further elucidate the potential specific role of DKD in modulating the association between pathways of declining kidney function and risk of T2D.^47^

While this MR study supports constructive understandings into the possible causal relationship between the inhibition of kidney function pathways and T2D risk in African populations, it is imperative to admit certain limitations. First, as with any data-based study, the risk of residual confounding cannot be completely controlled. Although we employed genetic instruments to reduce confounding by environmental and lifestyle considerations, unmeasured confounders may possibly still influence the observed associations.^48^ Secondly, the comparatively smaller sample size of African cohorts in relation to those of European ancestry may limit the statistical power of our analyses. MR studies generally mimic rather than replace randomized trials.^18^ The unique characteristics of genetic variants allow MR studies to assess the potential effect of lifelong low-dose exposure on desired clinical outcomes.^3^ In contrast, randomized trials generally evaluate the therapeutic effects of interventions at comparable doses over a relatively short period, typically ranging from a few months to five years.

The consistency of our findings across multiple sensitivity analyses, including MR-Egger regression, weighted median, and weighted mode methods, strengthens the robustness of our conclusions.^49^ The absence of significant pleiotropy, as indicated by the MR-Egger intercept and the symmetric distribution of effect estimates in the funnel plots (Figure S2), further supports the validity of our results.^50,51^ Additionally, the leave-one-out sensitivity analysis (Figure S1) and scatter plot of genetic associations (Figure S3) demonstrate that the observed associations are not driven by specific genetic variants or confounding factors. These findings collectively suggest that the observed associations between VEGFA and RHEB inhibition and increased T2D risk are robust and not influenced by outliers or pleiotropic effects.

The study balances well in examining the genetic susceptibility to T2D Risk in Africans even though it is pertinent to note the interplay of environmental and lifestyle factors.^3,24^ Further studies on the specified factors including diet, physical activity, and access to healthcare should explore their relationships with medication utilization and the heritability of T2D, with the aim of providing a comprehensive understanding of T2D risk in African groups.^52^ It is hopeful that by embracing a multifactorial approach that recognizes both genetic and environmental factors in T2D, researchers will be able to devise better preventive and management measures for T2D in Africa.

## Conclusion

The present work enlightens the following important research issue: some of medications for kidney diseases may increase T2D risk among Africans. Compared with preceding studies, it extracts African-specific genetic information and determines that certain pathways are associated with a higher T2D risk. This underscores the need for tailored treatment strategies in Africa. These findings spark a positive message of improved care. The elements of personalized medicine approaches may be adopted because of the factors that predispose individuals to T2D and the factors that contribute to chronic kidney disease as well. However, it is imperative that future studies, undertaken with a broader sample from the African region, utilize more sophisticated analytical tools. Several strategies that are classified under public health interventions multiply the influence T2D risk, especially those what advocate for healthy lifestyles and improved healthcare facilities. This, coupled with the pursuit of less expensive treatment modalities and increased medication availability, is critical for implementation. This study provides a clear knowledge about the problem deeply rooted in the African culture thus paving the way to find a proper treatment. This knowledge impacts positive changes in the recognition, treatment, and prevention of many diseases by healthcare providers, thus enhancing the quality of healthcare in Africa.

## Supplemental Material

sj-docx-1-sci-10.1177_00368504251338631 - Supplemental material for Genetically proxied inhibition of kidney function pathways and increased risk of type 2 diabetes in Africans: A Mendelian randomization study Supplemental material, sj-docx-1-sci-10.1177_00368504251338631 for Genetically proxied inhibition of kidney function pathways and increased risk of type 2 diabetes in Africans: A Mendelian randomization study by Abdoulaye Diawara, Mariam Traore, Oudou Diabaté, Christopher Kintu, Ali Awadallah Saeed, Julianah Ore Abiola, Cheickna Cisse, Kassim Traore, Mamadou Wele, Oyekanmi Nash, Seydou O. Doumbia, Talib Yusuf Abbas, Jeffrey G. Shaffer, Mahamadou Diakité, Segun Fatumo and Opeyemi Soremekun in Science Progress

## Abbreviations

ACE: angiotensin-converting enzyme
ADA: American Diabetes Association
ARB: angiotensin II receptor blocker
BMI: body mass index
CKD: chronic kidney disease
DKD: diabetic kidney disease
OAT: organic anion transporters
eGFR: estimated glomerular filtration rate
HbA1c: glycated hemoglobin
GFR: glomerular filtration rate
GWAS: genome-wide association study
MR: Mendelian randomization
IDF: International Diabetes Federation
LMICs: lower and middle-income countries
RCT: randomized controlled trial
SLC22A2: solute-like carrier family 22 members 2
CKDGen: chronic kidney disease genetics
VEGFA: vascular endothelial growth factor A
RHEB: Ras homolog enriched in brain
T2D: type 2 diabetes mellitus
SNP: single-nucleotide polymorphism
UK: Biobank (UKBB)
MVP: Million Veteran Program
AADM: The Africa America Diabetes Mellitus (AADM)
IVW: inverse variance weighted
VEGFR: vascular endothelial growth factor receptor
mTOR: mammalian target of rapamycin.
