# Supplementary material for: Genetically proxied inhibition of kidney function pathways and increased risk of type 2 diabetes in Africans: A Mendelian randomization study
Source: Sci Prog. 2025 Jun 17;108(2):00368504251338631. doi: 10.1177/00368504251338631 (PMC12174782; doi:10.1177/00368504251338631)
Supplement: sj-docx-1-sci-10.1177_00368504251338631 - Supplemental material for Genetically proxied inhibition of kidney function pathways and increased risk of type 2 diabetes in Africans: A Mendelian randomization study [file sj-docx-1-sci-10.1177_00368504251338631.docx]

**Supplementary material**

Table S1: Characteristics of genetic variants analyzed in MR study of kidney function drug targets and type 2 diabetes in Africa.

| Gene | SNPs | Effect  allele | other  allele | NSNPs | F | Median F (range) |
| --- | --- | --- | --- | --- | --- | --- |
| VEGFA | rs4714699 | T | C | **6** | 35.105 | **91.98577** |
|  | rs881858 | A | G |  | 181.413 |  |
|  | rs11758441 | T | C |  | 77.651 |  |
|  | rs9472141 | T | C |  | 109.788 |  |
|  | rs6458355 | A | G |  | 87.853 |  |
|  | rs9472144 | T | C |  | 96.118 |  |
| RHEB | rs6952398 | T | C | **9** | 38.081 | **136.843** |
|  | rs6967838 | T | C |  | 136.843 |  |
|  | rs2727564 | A | G |  | 51.208 |  |
|  | rs2374270 | A | C |  | 75.030 |  |
|  | rs73158180 | A | C |  | 152.917 |  |
|  | rs66497154 | T | C |  | 150.356 |  |
|  | rs10480300 | T | C |  | 213.189 |  |
|  | rs10265221 | T | C |  | 243.297 |  |
|  | rs13239239 | T | C |  | 76.2129 |  |
| CLDN14 | rs218642 | T | C | **4** | 34.363 | **34.024** |
|  | rs219793 | T | C |  | 37.039 |  |
|  | rs219788 | C | T |  | 33.686 |  |
|  | rs219771 | T | C |  | 30.085 |  |
| SLC22A2 | rs2297374 | T | C | **8** | 41.229 | **141.102** |
|  | rs2928035 | A | G |  | 80.496 |  |
|  | rs3127575 | T | C |  | 231.496 |  |
|  | rs316020 | A | G |  | 197.093 |  |
|  | rs76182445 | A | G |  | 175.509 |  |
|  | rs7757336 | T | G |  | 127.599 |  |
|  | rs316035 | T | C |  | 154.604 |  |
|  | rs3127590 | T | G |  | 94.225 |  |

Leave-One-Out Analysis

**
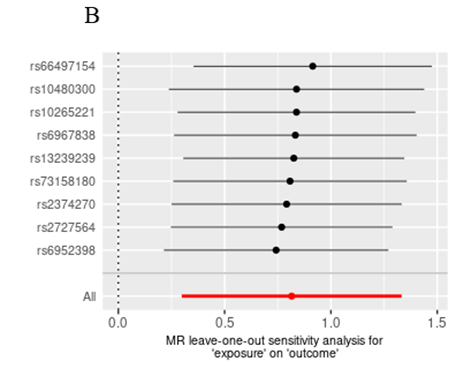

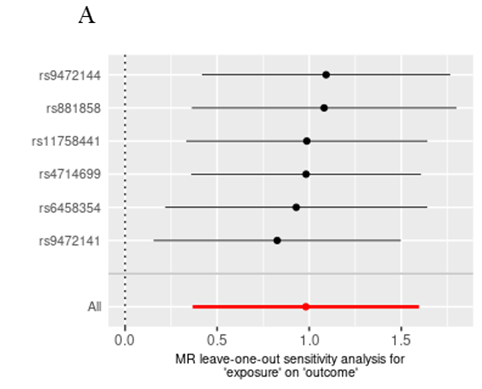
**

Figure S1 : MR Leave-One-Out Sensitivity Analysis for VEGFA(A) and RHEB (B)

*Leave-one-out sensitivity analysis for VEGFA and RHEB inhibition. Each bar represents the effect estimate when a single SNP is removed from the analysis, demonstrating the stability of the results.*

**Funnel Plot**


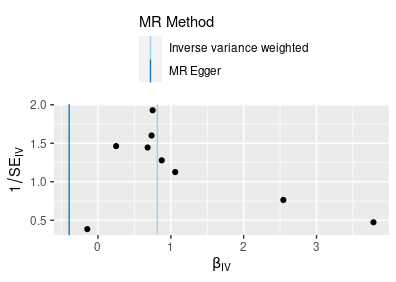
**
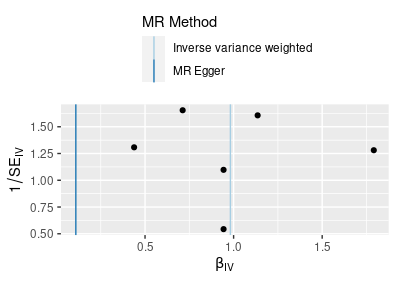
A B**

Figure S2: Funnel plot assessing potential pleiotropy in the MR analysis for VEGFA(A) and RHEB (B). The x-axis shows the effect estimates for each SNP, and the y-axis shows the precision (1/SE). The vertical line represents the IVW estimate, and the funnel shape indicates the expected distribution of effect estimates in the absence of pleiotropy.

Scatter Plot

**
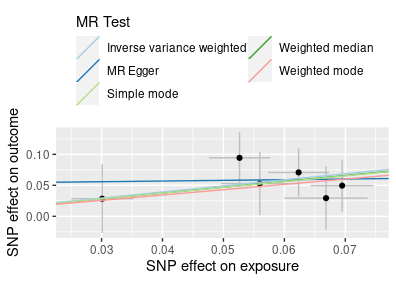
**
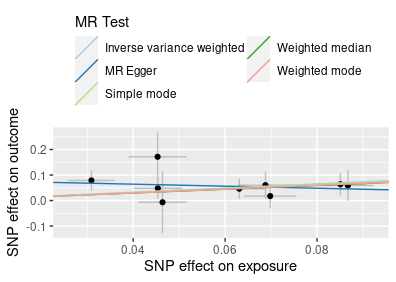
**A**  **B**

*Figure S3: Scatter plot of genetic associations for VEGFA and RHEB inhibition with T2D risk. Each point represents a single SNP, with the x-axis showing the SNP’s association with eGFR (exposure) and the y-axis showing its association with T2D (outcome). The solid line represents the IVW estimate, while the dashed lines represent the 95% confidence interval.*
